# Spectral domain-optical coherence tomography retinal biomarkers in choroidal neovascularization of multifocal choroiditis, myopic choroidal neovascularization, and idiopathic choroidal neovascularization

**DOI:** 10.1080/07853890.2021.1961015

**Published:** 2021-08-06

**Authors:** Rui Gao, Jingxue Ma, Zhengwei Zhang, Qingli Shang, Jialiang Duan

**Affiliations:** a Ophthalmology Department, Second Hospital of Hebei Medical University, Shijiazhuang City, Hebei province, China; b Ophthalmology Department, Nanjing Medical University Affiliated Wuxi Second Hospital, Wuxi City, Jiangsu Province, China

**Keywords:** Optical coherence tomography, multifocal choroiditis, choroidal neovascularization, myopic choroidal neovascularization, idiopathic choroidal neovascularization

## Abstract

**Objective:**

To use optical coherence tomography (OCT) to compare retinal biomarkers of choroidal neovascularization (CNV) secondary to multifocal choroiditis (MFC), myopic choroidal neovascularization (mCNV), and idiopathic choroidal neovascularization (ICNV) and to provide a basis for its clinical diagnosis and treatment.

**Methods:**

In this retrospective case study, patients admitted to the Second Hospital of Hebei Medical University between January 2018 and January 2021 who were initially diagnosed with CNV secondary to MFC, mCNV, and ICNV were categorized into groups, by disease, for analysis. Spectral domain-OCT (SD-OCT) was used to describe and measure the morphological characteristics of CNV lesions in each group. The retinal biomarkers of CNV in MFC, mCNV, and ICNV were compared.

**Results:**

Sixty-eight patients (71 eyes) were included and all eyes were diagnosed with active type 2 CNV. The MFC group had higher refraction than the ICNV group (P2 < 0.05). The choroidal thickness (CT) and CNV diameter of the MFC group were significantly greater than those of the mCNV group (P1 < 0.05). The number of eyes with sub-retinal fluids (SRF) and a “pitchfork sign” was significantly greater in the MFC group than in the mCNV group (P1 < 0.05). There was a significant difference only in CT) values between the MFC and ICNV groups (P2 < 0.001), but not in the other observation indicators (P2 > 0.05).

**Conclusions:**

OCT biomarkers, such as the diameter of the CNV, SRF, the “pitchfork sign,” and CT under CNV are useful in distinguishing CNV secondary to MFC from mCNV, which can allow the timely selection of treatment in some difficult cases. There were no differences between the MFC group and ICNV group except in refractive error, which indicates that some ICNV cases may be an early stage of a type of occult chorioretinitis. Long-term follow-up is needed for ICNV patients to confirm whether there is any potential inflammation.

**Key messages**
- Sometimes, it is difficult to separate MFC with CNV from myopic CNV and ICNV in clinical.
- OCT biomarkers, such as the diameter of the CNV, SRF, the “pitchfork sign,” and CT under CNV are useful in distinguishing CNV secondary to MFC from mCNV.
- There were no differences between the MFC group and ICNV group except in refractive error.

## Introduction

Multifocal choroiditis (MFC) is an idiopathic inflammatory disease characterized by punched-out or multiple yellowish white lesions that occur in the retinal pigment epithelium (RPE) and the choroidal capillary layer with little or no ocular inflammation [1]. Punctate inner choroidopathy (PIC) targets the same essential structures in a similar phenotypic manner and is treated in the same way as MFC, for which it is now considered part of the MFC spectrum by many researchers [2,3]. This inflammatory condition occurs predominantly in young (median age, 30 years), healthy, medium-to-high myopic (median refractive error of −7.00 dioptres [D]) women with no known associated systemic disease [4]. Clinically, patients may complain of decreased vision, floaters, photopsia, and temporal blind spots [1,5,6]. Choroidal neovascularization (CNV) secondary to MFC may significantly impact vision prognosis; its reported incidence oscillates between 22% and 69% [4].

As MFC occurs mainly in medium to highly myopic eyes, it may present with similar retinal characteristics to eyes with pathological myopia, such as a tilted optic disc, peripapillary atrophy, posterior staphyloma, and retinal pigment epithelial atrophy. Thus, in some cases, it is difficult to determine whether CNV is caused by MFC or myopia [7].

Furthermore, some early MFC cases may present as a single lesion with minimal vitreous cells [7]. In addition, some hyperfluorescent patches seen on indocyanine angiography (ICGA) in PIC patients may have a normal appearance with a fundus examination and spectral-domain optical coherence tomography (SD-OCT) [8]. It is possible that some patients diagnosed with idiopathic choroidal neovascularization (ICNV) initially may develop MFC/PIC in subsequent follow-up [9]. The conventional treatment method for mCNV and ICNV involves intravitreal anti-vascular endothelial growth factor (VEGF) therapy. In addition to anti-VEGF therapy, CNV secondary to MFC also require the use of systemic or local immunosuppressive therapies in most cases [4].

For the above reasons, being able to distinguish between CNV secondary to MFC, mCNV, or ICNV is important for clinical management. Discrimination may be difficult with traditional methods [7]. Thus, in this study, we aimed to analyze the difference between CNV secondary to MFC, mCNV, and ICNV by observing and comparing retinal biomarkers on OCT.

## Materials and methods

**Figure 1.**
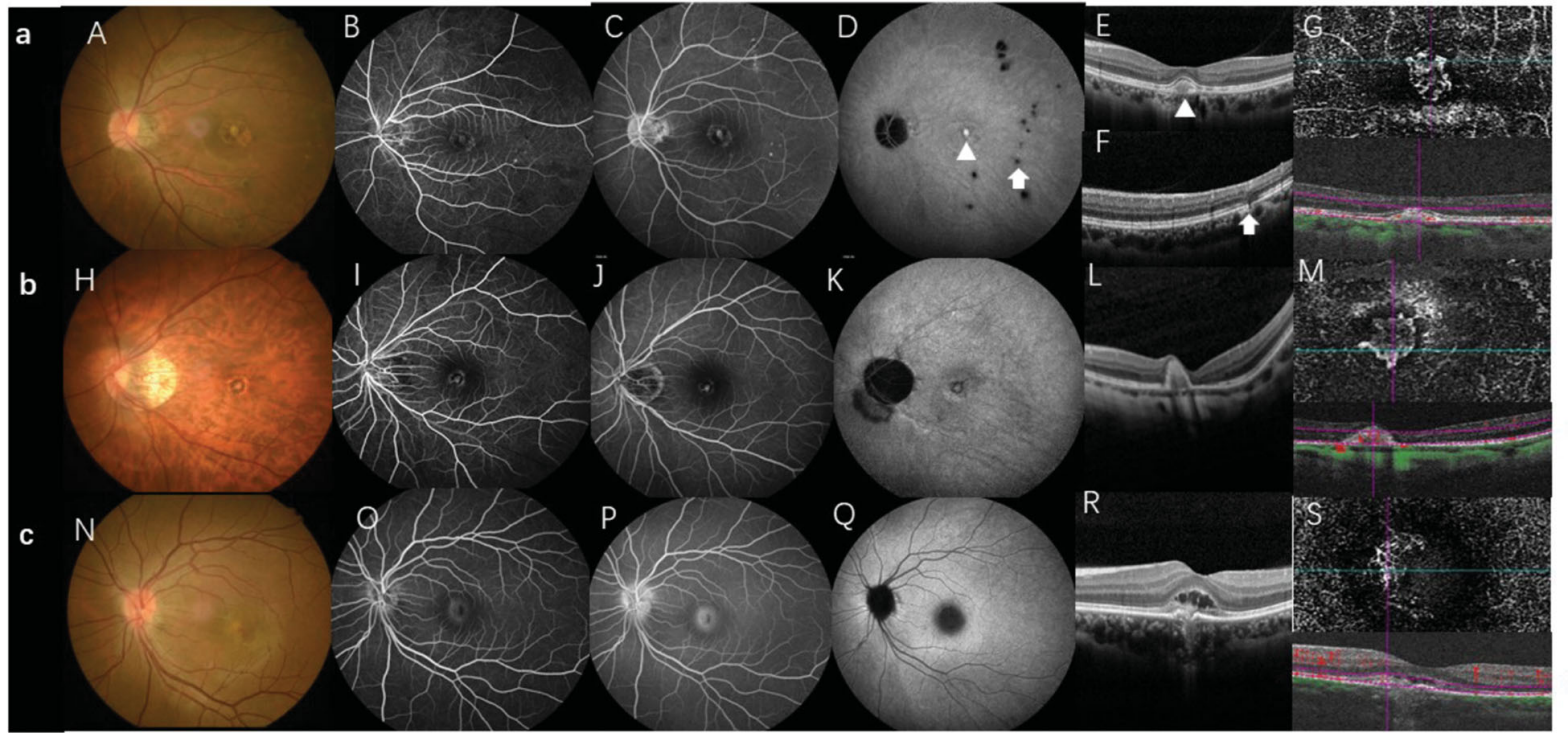
Multi-model imaging of MFC with CNV, mCNV, and ICNV. Line a: MFC with CNV. (A) The colour fundus photograph shows multiple small yellowish lesions and a few scattered atrophic spots. (B, C) FFA showing early hyperfluorescence and late leakage is the typical characteristic of type 2 CNV. (D) The late phases of ICGA show macular lesion with hyperfluorescence and there are multiple hypofluorescence spots which are greater in number and larger than those observed using fundus photography and FFA. (E) OCT scans corresponding to red arrowheads in image D demonstrate subretinal hyperreflective material overlying the RPE. (F) OCT scans corresponding to the green arrowhead in image D demonstrate that the photoreceptor layer around the lesion is lost; choroidal hyperreflectivity is well demonstrated here. (G) OCTA revealed detectable flow above the RPE. Line b: mCNV. (H) Colour fundus photograph of the left eye shows a tilted optic disc, posterior staphyloma, peripapillary atrophy, leopard fundus, and a greyish lesion surrounded with a hyperpigmented border at macular. (I, J) Early phase of the fluorescein angiogram showing very small, well-defined hyperfluorescence with minimal leakage in the late phases. (K) The late phases of ICGA show hyperfluorescence and a lacquer crack around the CNV. (L) OCT showing a hyper-reflective lesion corresponding to myopic CNV and a thinning choroidal layer. (M) OCTA shows detectable flow above the RPE. Line c: Typical ICNV. (N–R) The colour fundus photograph, FFA, and ICGA OCT of the ICNV showed there were only the typical characteristics of type 2 CNV. (S) The OCTA of the ICNV revealed detectable flow above the RPE. CNV: choroidal neovascularization; MFC: multifocal choroiditis; ICGA: indocyanine angiography; OCT: optical coherence tomography; FFA: fundus fluorescein angiography; mCNV: myopic choroidal neovascularization; ICNV: idiopathic choroidal neovascularization; RPE: retinal pigment epithelium; OCTA: optical coherence tomography angiography.

The study protocols were approved by the Ethical Committee of the Affiliated Second Hospital of Hebei Medical University. The requirement for written informed consent was waived owing to the retrospective nature of the study. The medical files of patients initially diagnosed with CNV caused by MFC, myopic, and idiopathic CNV who were referred to the Second Hospital of Hebei Medical University were retrospectively reviewed. CNV diagnosis was confirmed by the presence of early phase hyperfluorescence with late-phase leakage on fundus fluorescein angiography (FFA). ICGA, optical coherence tomography angiography (OCTA) and SD-OCT were performed to assist in the diagnosis of CNV [10]. Patients were included in the MFC group if they had idiopathic, multiple yellow-white choroidal lesions or punched-out lesions visible on colour fundus photography, and the CNV features mentioned above (Figure 1) [6]. In our study, we did not separate PIC from MFC. Patients with a refractive error of −6 D or worse, along with fundus changes and the typical manifestations of CNV and without other fundus lesions [11] (Figure 1), were included in the mCNV group. Patients were added to the ICNV group if the cause of CNV could not attributed to any ocular or systemic disease [10] (Figure 1). The exclusion criteria were as follows: (1) patients with any ocular diseases other than pathological myopia of MFC/PIC; (2) patients whose diagnosis was unclear and difficult to classify; (3) patients who had undergone previous treatment and had old CNV lesions; (4) patients who had systemic inflammation or an infectious disease; and (5) poor image quality that prevented recognizing the disease.

**Figure 2.**
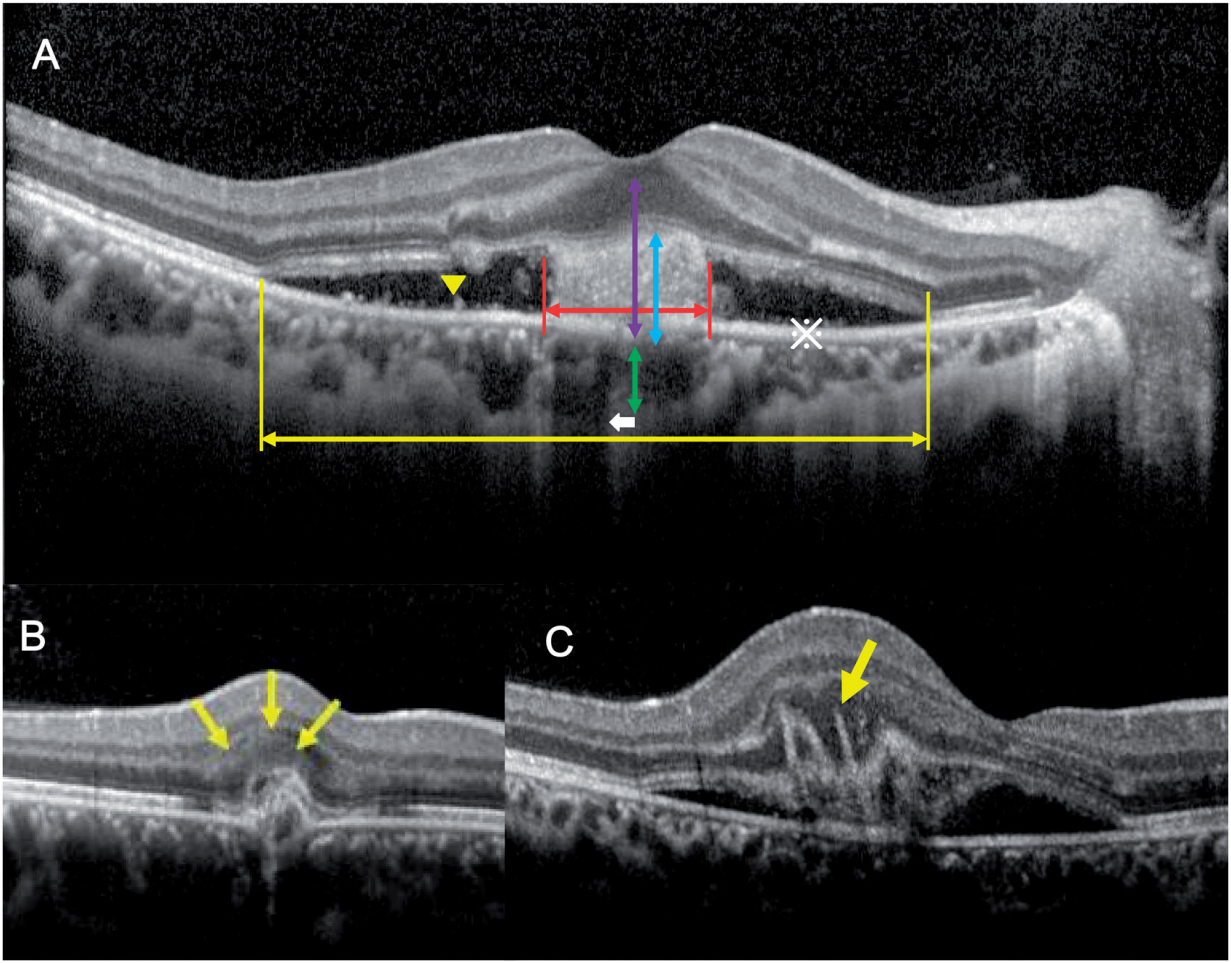
Schematic diagram of the OCT biomarker and measurement of CNV lesions. (A) The yellow double arrow indicates the disrupted ellipsoid zone length and the red double arrow indicates the CNV diameter. The purple double arrow indicates the height of entire lesion, the blue double arrow indicates the height of the CNV, and the green double arrow indicates the choroidal thickness. The yellow arrow-head indicates the hyperreflective dots and the asterisk (※) indicates the area with a low reflected signal, i.e. the subretinal fluid. (B) The yellow arrow indicates a subretinal hyperreflective exudate. C. The yellow arrow indicates the "pitchfork sign." CNV: choroidal neovascularization; OCT: optical coherence tomography.

The baseline characteristics of patients and the tomographic features of CNV on SD-OCT (Heidelberg Engineering, Heidelberg, Germany) were recorded. FFA, ICGA (Heidelberg Engineering), and OCTA (Carl Zeiss Meditec, Germany) images were obtained. Diagnosis and classification of CNV were performed by two vitreoretinal specialists (Q.S. and J.D.). We intended to cover all morphological features of CNV known in the literature at the time of protocol establishment. The quantitative parameters included the choroidal thickness [12] (CT) beneath the CNV, the disrupted ellipsoid zone length, [13] the height of the entire lesion defined as the distance between the Bruch membrane and the innermost layer of the disrupted retina, the diameter and height of the CNV, [14] and the thickness of the central macular retinal thickness (CMT) [15]. Qualitative indicators including the CNV location [16] (if the CNV was positioned 1–199 μm from the centre of the area of foveal (as shown by OCT), it was considered foveal-juxtafoveal; if the CNV was positioned at 200 μm or further it was considered extrafoveal), the presence of intraretinal cystic lesions, [17] the presence of sub-retinal fluids (SRF), [17] the presence of subretinal hyperreflective exudation (SHE), [17] the fuzziness of the border of the hyper-reflective CNV lesion, [16] the presence of hyperreflective dots and a shadowing effect towards the choroid, [18] the presence of a “pitchfork sign”, and the presence of focal choroidal excavation [19] (Figure 2).

All data in this study were analyzed using SPSS (version 21) statistical software. Continuous, normally distributed data are expressed as the mean ± standard deviation and were compared using a group *t*-test. Non-normally distributed data are presented as the median (1st quartile–3rd quartile) and were compared using the Wilcoxon rank-sum test. Numeric data are expressed as the rate and composition ratio, and comparisons were performed using the Chi-square test. Differences were considered statistically significant at *p* < .05.

## Results

### Patients

We enrolled 68 patients (71 eyes) in this study, all of whom were diagnosed with active CNV. Forty-one eyes of 38 patients belonged to the MFC group, and most patients had unilateral CNV (*n* = 34); four cases were diagnosed with bilateral CNV (three patients had bilateral active CNV; one patient who had an active CNV lesion in one eye and an inactive CNV lesion in the other eye was excluded). There were 32 women (84.2%) and six men (15.8%). The mean age of the patients was 40 ± 11.25 years (range, 18–80 years), and the mean refractive error was −8D. A total of 19 eyes of 19 patients were included in the mCNV group, including 12 women (63.2%) and seven men (36.8%). The average age was 42.68 ± 11.97 years (range, 17–67 years) and the median refractive error was −9D. Of the 11 patients with ICNV, seven patients were women (63.6%) and four patients were men (36.4%). The mean age was 33.82 ± 8.01 years (range, 23–46 years), and the median refractive error was −3.5D (Table 1).

**Table 1.** Comparison between the MFC secondary to CNV, mCNV, and ISCNV groups.

	MFC-CNV	mCNV	ISCNV	P1*	P2*
Gender, No. (%)				0.099	0.201
Men	6 (15.8%)	7 (36.8%)	4 (36.4%)		
Women	32 (84.2%)	12 (63.2%)	7 (63.6%)		
Age, years	40.0 ± 11.3	42.7 ± 12.0	33.8 ± 8.0	0.390	0.106
Refractive error (D)	−8 (4.875, 10)	−9 (7.5, 11)	−3.5 (1.5, 4.5)	0.478	<0.001
CT (µm)	152 (91, 224)	69 (55, 94)	263 (227, 325)	<0.001	<0.001
Disrupted ellipsoid zone length (µm)	1825 (1206, 2484)	1506 (817, 1999)	1961 (1347, 2652)	0.267	0.509
Entire lesion height (µm)	318 (236, 405)	295 (226, 344)	339 (208, 427)	1.000	0.946
CNV diameter (µm)	1090 (824, 1621)	828 (510, 1002)	947 (593, 1168)	0.026	0.100
CNV height (µm)	223 (159, 312)	159 (130, 249)	223 (149, 288)	0.096	0.937
CMT (µm)	311 (250, 371.5)	311 (278, 359)	344 (284, 363)	0.838	0.382
CNV location				0.416	1.000
Foveal-juxtafoveal	34 (82.9%)	18 (94.7%)	10 (90.9%)		
Extrafoveal	7 (17.1%)	1 (5.3%)	1 (9.1%)		
Intraretinal cystic				0.225	0.735
Yes	22 (53.7%)	7 (36.8%)	7 (63.6%)		
No	19 (46.3%)	12 (62.3%)	4 (36.4%)		
SRF				0.003	0.253
Yes	33 (80.5%)	8 (42.1%)	7 (63.6%)		
No	8 (19.5%)	11 (57.9%)	4 (36.4%)		
SHE				0.515	1.000
Yes	30 (73.2%)	16 (84.2%)	8 (72.7%)		
No	11 (26.8%)	3 (15.8%)	3 (27.3%)		
Fuzzy border				1.000	0.664
Yes	33 (80.5%)	16 (84.2%)	10 (90.9%)		
No	8 (19.5%)	3 (15.8%)	11 (9.1%)		
Hyperreflective dots				0.781	0.463
Yes	21 (51.2%)	9 (47.4%)	7 (63.6%)		
No	20 (48.8%)	10 (52.6%)	4 (36.4%)		
Shadowing effect				0.585	0.193
Yes	39 (95.1%)	17 (89.5%)	9 (81.8%)		
No	2 (4.9%)	2 (10.5%)	2 (18.2%)		
Pitchfork sign				0.007	0.291
Yes	16 (39.0%)	1 (5.3%)	2 (18.2%)		
No	25 (61.0%)	18 (94.7%)	9 (81.8%)		
FCE				0.168	1.000
Yes	5 (12.2%)	0 (0.0%)	1 (9.1%)		
No	36 (87.8%)	19 (100.0%)	10 (90.9%)		

P1: Comparison between the CNV secondary to MFC and mCNV groups.

P2: Comparison between the CNV secondary to MFC and ICNV groups.

CNV: choroidal neovascularization; MFC: multifocal choroiditis; ICNV: idiopathic choroidal neovascularization; CT: choroidal thickness; CMT: central macular retinal thickness; SRF: subretinal fluids; SHE: subretinal hyperreflective exudation; FCE: focal choroidal excavation.

### Comparison between CNV secondary to MFC and mCNV

**Figure 3.**
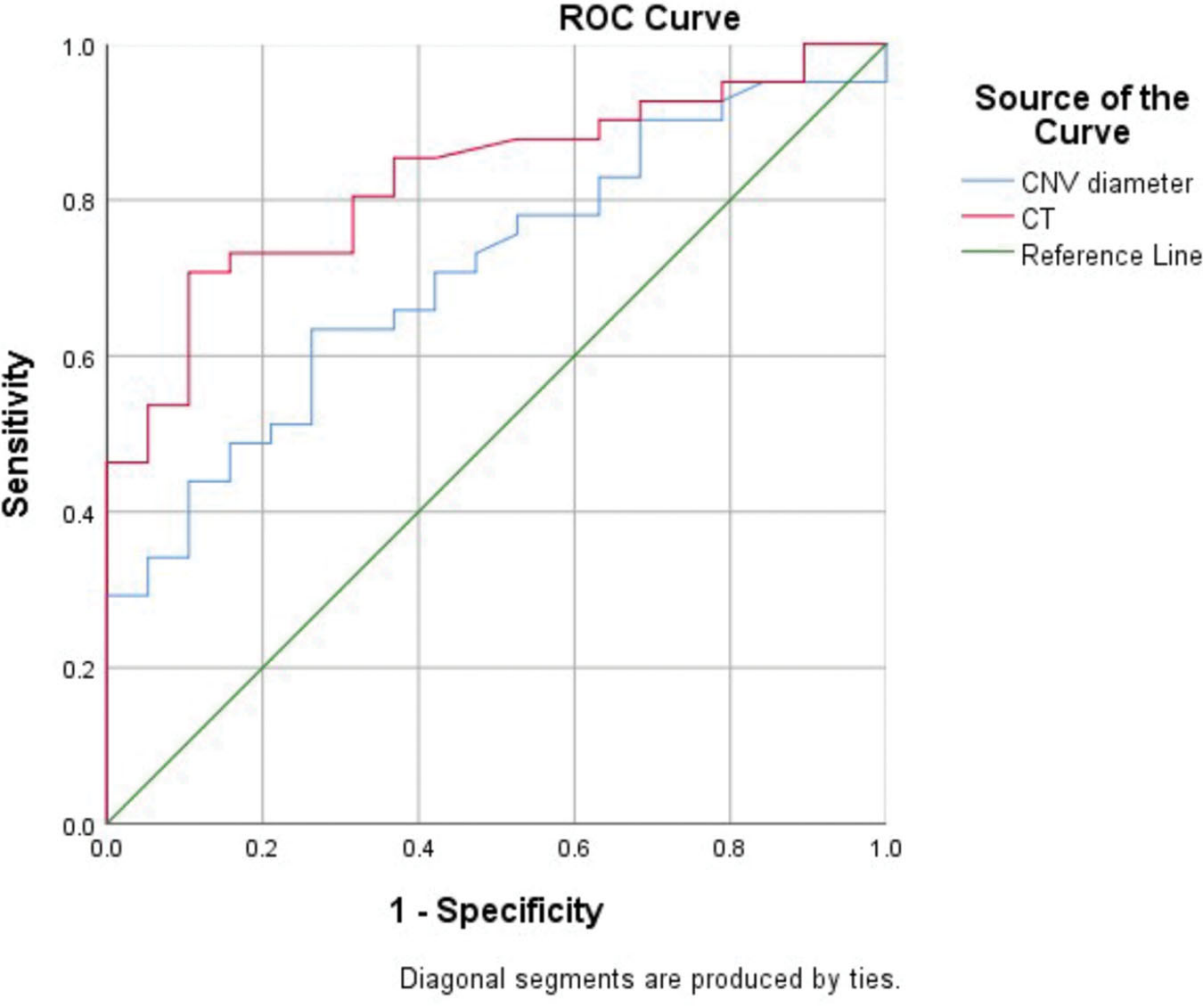
The ROC curve of the CNV diameter and CT value in distinguishing CNV secondary to multifocal choroiditis and myopic CNV. ROC: receiver operator characteristic; CNV: choroidal neovascularization; CT: choroidal thickness.

There were no statistical differences in the clinical characteristics of sex, age, or refractive error between the MFC and mCNV groups (Table 1). We compared the OCT biomarkers of CNV lesions in the two groups. Among the quantitative indicators, CT beneath the CNV and the CNV diameter in the MFC group were significantly higher than those in the mCNV group, and the differences were statistically significant (P1 < 0.05). Next, the receiver operator characteristic (ROC) curve was drawn to determine the cut-off level of the CT and CNV diameter for differencing CNV secondary to MFC from mCNV (Figure 3). According to the Youden index, the optimal critical value of the CT was 104 µm, and the corresponding diagnostic sensitivity, specificity, accuracy, and Youden index were 70.7%, 89.5%, 82.7%, and 0.602%, respectively. The optimal critical value of the CNV diameter was 888.5 µm and the corresponding diagnostic sensitivity, specificity, accuracy, and Youden index were 63.4%, 73.7%, 70.7%, and 0.371%, respectively. Considering the qualitative indicators, the number of patients with SRF or a “pitchfork sign” on OCT in the MFC group was greater than that in the mCNV group, and the difference was statistically significant (P1 < 0.05). There were no significant differences in the remaining observation indexes between the two groups (P1 > 0.05) (Table 1).

### Comparison between CNV secondary to MFC and ICNV

There were no statistical differences in the clinical characteristics of sex and age in the two groups (Table 1). Patients with CNV secondary to MFC had more severe myopia (P2 < 0.001). The CT values of the OCT manifestations were statistically significant between the two groups (P2 < 0.001). However, there were no statistical differences between the two groups regarding the CT beneath the CNV, the CNV diameter, the percent of patients with SRF, or a “pitchfork sign” on OCT and other biomarkers (P2 ≥ 0.1).

## Discussion

**Figure 4.**
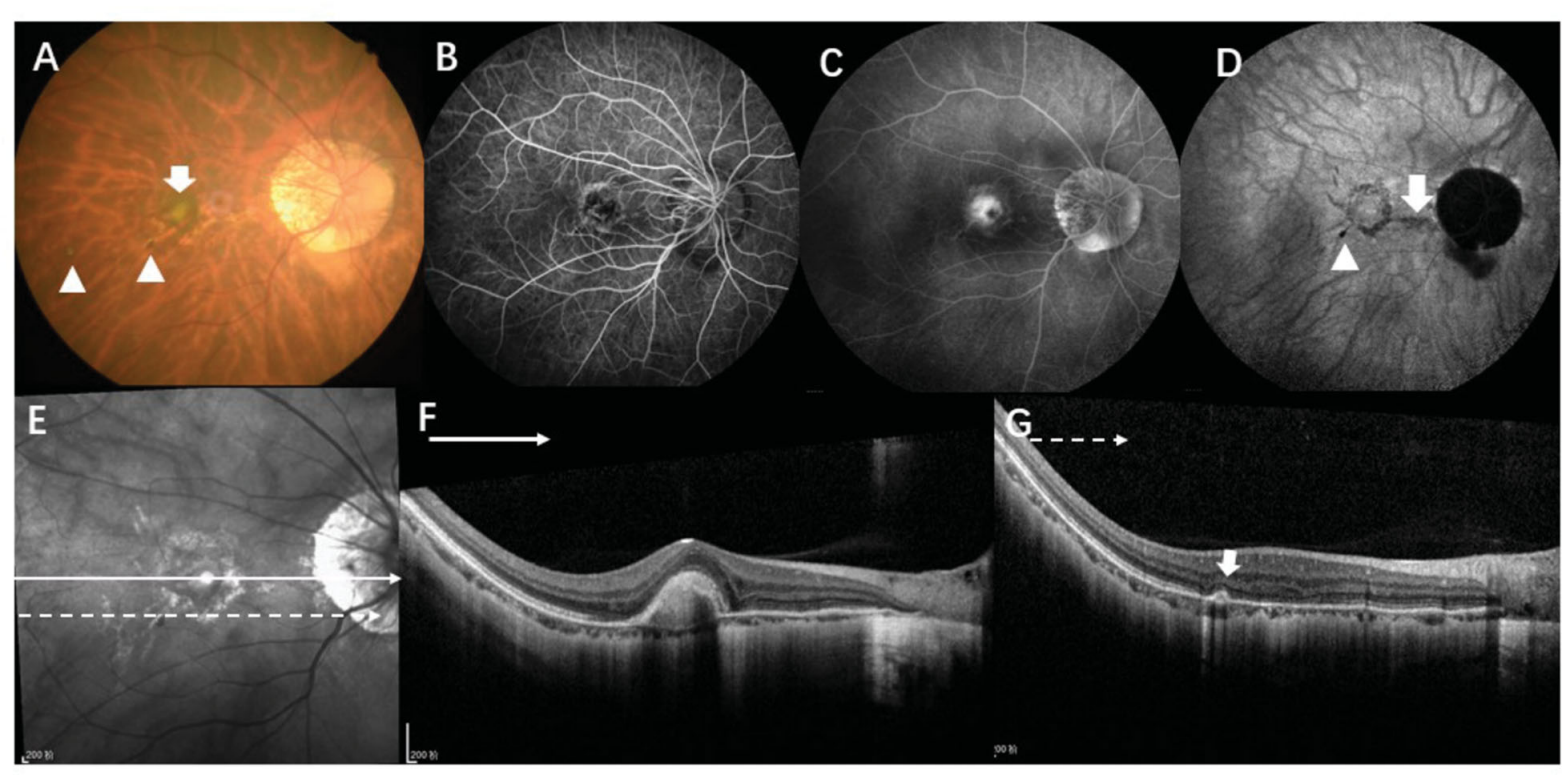
Multimodal images of a 36-year-old woman with high myopia (-9D in both eyes) where it was difficult to determine the aetiology of CNV. (A) A colour fundus photograph of the right eye shows typical high myopia changes including peripapillary atrophy, leopard fundus, and posterior staphyloma. In addition, there is a yellowish lesion in the macular region (green arrow) corresponding to CNV as well as two very small lesions (yellow arrow) in the infer-temporal retina. (B, C) FFA showing early hyperfluorescence and late leakage. (D) Late phase of ICGA showing a lacquer crack underneath the CNV lesion (red arrow) and a hyporeflective spot which corresponds to a very small lesion which can be seen in the fundus photograph (yellow arrow). (E, F) OCT scan of the CNV lesions (green line) shows high reflectance on the RPE and rupture of the ellipsoid zone. (G) OCT scan of the lesion seen in the fundus photograph and ICGA (yellow arrow) shows sub-RPE hyperreflexia with choroidal hyper-transmission and adjacent ellipsoid zone disruption which is similar to OCT findings of an inflammation lesion caused by MFC/PIC. CNV: choroidal neovascularization; MFC; multifocal choroiditis; OCT: optical coherence tomography; FFA: fundus fluorescein angiography; RPE: retinal pigment epithelium.

CNV is a pathological change that occurs in a variety of retinal and choroidal diseases and seriously affects patients’ vision and reduces their quality of life [10]. Previous research has found that the most common aetiologies of CNV among young patients are pathologic myopia, inflammation, and ICNV [10,20]. Usually, CNV secondary to MFC can be differentiated from mCNV lesions by the presence of vitreous cells and via fundus examination according to the characteristic yellow-white or punched-out lesions that present with early hyperfluorescence and late fluorescence leakage in FFA in the same or the fellow eye [11]. However, MFC/PIC also occurs in patients with high myopia; since both conditions have similar characteristics, it is difficult determine the cause of CNV under some circumstances by conventional examinations like FA or ICGA [7] (Figure 4). OCTA may be helpful to differentiate CNV or inflammation lesions, but not to distinguish the aetiology from inflammatory CNV or non-inflammatory CNV [11].

ICNV is a single and focal CNV that occurs in adults younger than 50 years of age without any primary ocular or systemic disease [21] (Fig. 4). However, early-on, patients with MFC/PIC may only have one lesion, [22] making it difficult to distinguish it from ICNV. CNV secondary to MFC may require immunosuppressive therapy in addition to anti-VEGF therapy in most cases, which is different from the treatment for mCNV and ICNV. In this study, we aimed to identify some OCT biomarkers to distinguish the three types of CNV.

A total of 68 patients were included, and the proportion of female patients with CNV was 70.6% (i.e. more than 50%), which was consistent with the results of previous studies [9]. The CT beneath the CNV lesions of patients affected by MFC with CNV and mCNV (the medians were 152 um and 69 um, respectively) was compared in this study. We found that the CT was thicker in the former group than in the latter. CT is affected by many factors, such as age, axial length, and female sex [23]. However, in this study, there were no statistically significant differences in age or sex distributions between the MFC and the mCNV groups. Although our study lacked data on the axial length of patients, the refractive error, which is closely related to the axial length, was not statistically different between the two groups. Therefore, we speculated that the difference in CT between the two groups was due to different aetiologies of CNV, and the inflammation of the CNV in MFC patients might cause thickening of the choroid. Giuffre et al. [12] retrospectively analyzed CT at the same location in patients with CNV secondary to MFC/PIC and mCNV. Their results showed that CT beneath inflammatory CNV significantly increased at baseline and decreased after therapy, which they called the “sponge sign”. Conversely, no significant CT changes were observed in eyes with mCNV. The authors hypothesized that increased release of inflammatory mediators may cause choroidal vessel dilation leading to choroidal thickening.

The diameter of CNV secondary to MFC was larger than that of mCNV, and more MFC patients showed SRF manifestations. This phenomenon may be related to the different pathogeneses of the two types of CNV. Although the pathogenesis of mCNV is not yet fully understood, most authors agree with the mechanical and heredodegenerative theories, which states that fissures in the RPE–Bruch’s membrane–choriocapillaris complex are caused by the elongation of myopic eyes [24]. The presence of lacquer cracks has been found to be associated with a higher risk of mCNV. In addition, large myopic conus and patchy atrophy, where choroidal capillaries are missed, are thought of as the precursor lesions of mCNV. This leads to a decrease in choroid perfusion and choroid ischaemia, which increases the vascular growth factor and CNV development [11] Some authors believe that this leads to low mCNV activity [18], which may also explain its smaller diameter. Retinal thinning and weakening of choroidal microcirculation may be the reasons for the relatively low incidence of SRF in contrast with inflammatory CNV. Most cases of inflammatory CNV are type 2, in which focal inflammation occurs; the inflammatory antigen will be deposited at the area of Bruch’s membrane and trigger the subsequent focal inflammatory response. This causes the rupture of Bruch’s membrane and proliferation of granulation tissue into the subretinal space [25]. A previous study found neovascular CNV bridging in inflammatory CNV, which may be the reason for its larger diameter [25]. The leakage of the neovascular complex may be related to the lesion itself, compromising the function of the retinal pigment epithelium, the health of the choroid, and the integrity of outer retina [26]. MFC is an inflammatory disease occurring in the RPE and inner choroidal that involves the outer retina; thus, it is not difficult to understand that patients with CNV secondary to MFC are more likely to show SRF on OCT.

The “pitchfork sign” is a concept proposed by Hoang et al. [27] who found that the OCT images of patients with CNV secondary to MFC/PIC revealed distinctive, multiple finger-like projections which extended from the active CNV into the outer retina. Moreover, a previous study by Giuffre [12] showed that 36% of patients with CNV secondary to MFC had this characteristic. The results of our study showed that 16 patients (39.0%) in the MFC group showed the “pitchfork sign”, while only one patient (5.3%) in the mCNV group did; the difference between the two groups was statistically significant (*p* < .05). The pathological features of the “pitchfork sign” are still unclear; Hoang et al. [27] speculated that it may be related to the lesions which progress from inflammatory subretinal deposit lesions to neovascularization lesions. This may explain why there were more patients who had the “pitchfork sign” in the MFC group which the CNV is secondary from inflammatory disease than mCNV group.

**Figure 5.**
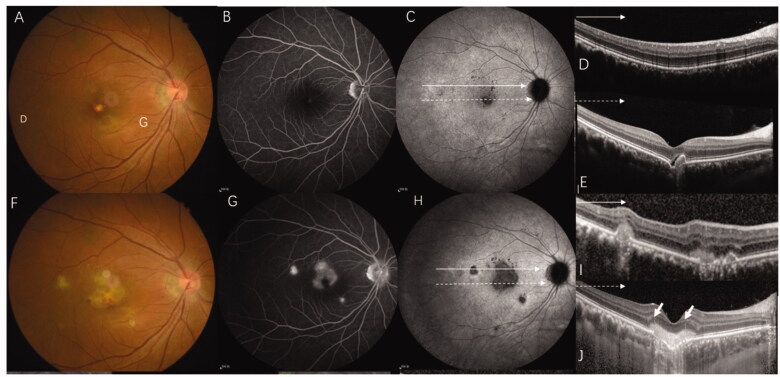
This 36-year-old women with myopia (-5 D in her right eye) complained of decreased visual acuity in her right eye for 5 days and was diagnosed with ICNV via multimodal imaging at her first visit. However, after being treated with anti-VEGF four times and after 6-months of follow-up, the final diagnosis was CNV secondary to MFC. (A–E) Multimodal image of the right eye, which was diagnosed with ICNV at the first visit. (A, B) The fundus photograph and FA only show the CNV lesion. (C) ICGA shows multiple hypofluorescence in the late phases. (D, E). OCT shows normal at superior part of macular (green line) and a CNV legion (yellow line). (F, G) Multimodal image of the same patient at the six-month follow-up. (F–H) The fundus photograph, FA, and ICGA show multiple new lesions. (I) The OCT scan of the same layer with figure D after 6 months shows a well-demarcated dome-shaped homogenous lesion (red arrow) and other new lesions with choroidal hyperreflectivity. (G) The CNV becomes larger and the "pitchfork sign" (yellow pointed arrow) is visible. Thus, the final diagnosis was CNV secondary to MFC. CNV: choroidal neovascularization; MFC: multifocal choroiditis; ICGA: indocyanine angiography; OCT: optical coherence tomography; FFA: fundus fluorescein angiography.

Compared with the ICNV group, the results of the MFC group only showed statistical differences in the refractive error and CT, while there were no statistical differences in the other morphological indicators. However, the choroid thickness is strongly influenced by the dioptre; therefore, it is hard to determine whether the difference between the two group is related to aetiology. Partial correlation analysis indicated no correlation between the two aetiologies and CT (*p* = .094). There are many similarities between CNV secondary to MFC and ICNV. Interestingly, one of the patients in this study was initially diagnosed with ICNV and was found to have a yellow-white lesion that appeared as an MFC in the ipsilateral eye after six-months of follow-up (Figure 5). A previous study found that four in 58 patients first diagnosed with ICNV developed inflammatory chorioretinal disease in the ipsilateral or contralateral eye during the follow-up period [9] In addition, relevant research found that nearly 60% of ICNV patients had hypofluorescence or hyperfluorescence on ICGA, which indicated that latent choroidal lesions may be present in ICNV patients [9].

Since this was a retrospective study, the inherent biases of this type of protocol cannot be neglected. First, some information, such as axial length was lacking owing to the retrospective nature of this study. Second, the follow-up data of patients were not collected and analyzed. And third, the relatively small number of patients in the MCNV and ICNV groups resulted in a certain degree of error in the results.

In conclusion, OCT biomarkers, such as the diameter of the CNV, SRF, the “pitchfork sign”, and the CT under CNV are useful in distinguishing CNV secondary to MFC from mCNV, which may help to decide on the proper treatment in a timely manner in difficult cases. Aside from refractive error, there were no differences between the MFC group and ICNV group, which indicates that some ICNV cases may be the early stage of chorioretinitis. Long-term follow-up is needed for ICNV patients in order to confirm whether there is any potential inflammation.

## Data Availability

All data that support the findings of this study are available from the corresponding author upon request.
